# Determining the Cause of Death Among Children Hospitalized With Respiratory Illness in Kenya: Protocol for Pediatric Respiratory Etiology Surveillance Study (PRESS)

**DOI:** 10.2196/10854

**Published:** 2019-01-10

**Authors:** Henry N Njuguna, Sherif R Zaki, Drucilla J Roberts, Corinne L Fligner, M Kelly Keating, Emily Rogena, Edwin Walong, Andrew K Gachii, Elizabeth Maleche-Obimbo, Grace Irimu, John Mathaiya, Noelle Orata, Rosemarie Lopokoiyit, Jackson Maina, Gideon O Emukule, Clayton O Onyango, Stella Gikunju, Collins Owuor, Peter Kinuthia, Milka Bunei, Barry Fields, Marc-Alain Widdowson, Joshua A Mott, Sandra S Chaves

**Affiliations:** 1 Division of Global Health Protection Centers for Disease Control and Prevention Atlanta, GA United States; 2 Infectious Disease Pathology Branch Centers for Disease Control and Prevention Atlanta, GA United States; 3 Department of Pathology Massachusetts General Hospital Boston, MA United States; 4 University of Washington Seattle, WA United States; 5 University of Nairobi Nairobi Kenya; 6 Kenyatta National Hospital Nairobi Kenya; 7 Influenza Program Centers for Disease Control and Prevention Nairobi Kenya; 8 Division of Global Health Protection Centers for Disease Control and Prevention Nairobi Kenya; 9 IHRC, Inc Nairobi Kenya; 10 Influenza Division Centers for Disease Control and Prevention Atlanta, GA United States

**Keywords:** cause of death, pneumonia, etiology, infectious disease, postmortem, mortality, respiratory illness

## Abstract

**Background:**

In sub-Saharan Africa, where the burden of respiratory disease–related deaths is the highest, information on the cause of death remains inadequate because of poor access to health care and limited availability of diagnostic tools. Postmortem examination can aid in the ascertainment of causes of death. This manuscript describes the study protocol for the Pediatric Respiratory Etiology Surveillance Study (PRESS).

**Objective:**

This study protocol aims to identify causes and etiologies associated with respiratory disease–related deaths among children (age 1-59 months) with respiratory illness admitted to the Kenyatta National Hospital (KNH), the largest public hospital in Kenya, through postmortem examination coupled with innovative approaches to laboratory investigation.

**Methods:**

We prospectively followed children hospitalized with respiratory illness until the end of clinical care or death. In case of death, parents or guardians were offered grief counseling, and postmortem examination was offered. Lung tissue specimens were collected using minimally invasive tissue sampling and conventional autopsy where other tissues were collected. Tissues were tested using histopathology, immunohistochemistry, and multipathogen molecular-based assays to identify pathogens. For each case, clinical and laboratory data were reviewed by a team of pathologists, clinicians, laboratorians, and epidemiologists to assign a cause of and etiology associated with death.

**Results:**

We have enrolled pediatric cases of respiratory illness hospitalized at the KNH at the time of this submission; of those, 14.8% (140/945) died while in the hospital. Both analysis and interpretation of laboratory results and writing up of findings are expected in 2019-2020.

**Conclusions:**

Postmortem studies can help identify major pathogens contributing to respiratory-associated deaths in children. This information is needed to develop evidence-based prevention and treatment policies that target important causes of pediatric respiratory mortality and assist with the prioritization of local resources. Furthermore, PRESS can provide insights into the interpretation of results using multipathogen testing platforms in resource-limited settings.

**International Registered Report Identifier (IRRID):**

DERR1-10.2196/10854

## Introduction

### Background

Pneumonia is a leading cause of death among children aged <5 years [1], and approximately 50% of this burden is borne by children in sub-Saharan Africa [2]. Nonetheless, in most resource-limited countries, the relative contribution of different pathogens leading to respiratory illness-related deaths remains largely unknown. Understanding the etiologies of respiratory illness-related deaths is key to targeting resources and informing policies for the treatment and prevention of respiratory illnesses. In resource-limited settings, information on the cause of death is determined through clinical diagnosis prior to death or, more frequently, verbal autopsy [3] because many deaths occur outside hospitals. The introduction and expansion of vaccination against major causes of childhood respiratory disease–related mortality, such as measles, *Streptococcus pneumoniae* and *Haemophilus influenzae B*, over the past decade and the availability of highly active antiretroviral treatment (HAART) [4] may have changed the relative roles of different etiologies of respiratory disease–related mortality in sub-Saharan Africa. Moreover, with improved multipathogen diagnostic techniques, studies have suggested a more substantial role of respiratory viruses in severe acute respiratory illnesses in young children [5-8].

Postmortem examination and laboratory testing of tissue specimens can aid in improving the ascertainment of causes of death [9]. However, few clinical postmortem examinations are currently performed [10], especially in sub-Saharan Africa where the respiratory disease burden is the highest [11]; this is partly attributed to a lack of resources and inadequate technical capacity to perform detailed postmortem examinations. Moreover, the poor understanding of the utility and practice of conventional autopsy allows for misinformation and stigma among patients and clinicians alike, hampering informed consent, which is required for the procedure. Families can be reluctant to consent to autopsy fearing that the bodies of their loved ones will be disfigured [12].

This study describes the methodology for the Pediatric Respiratory Etiology Surveillance Study (PRESS), which was conducted prospectively to investigate the cause of death and associated microbial etiologies among children (age 1-59 months) hospitalized for respiratory illness in Kenya. For children who died, we undertook a postmortem evaluation of gross pathology and histopathological findings and implemented multipathogen diagnostic and immunohistochemical procedures to identify pathogens potentially associated with death. Furthermore, we compared minimally invasive tissue sampling techniques (MITS) with conventional autopsy procedures to obtain lung tissue specimens.

### Research Objectives

The primary research goals of PRESS are to (1) describe the leading etiologies of pediatric respiratory disease–related deaths in a tertiary care hospital in Kenya, a low-middle-income country and (2) evaluate methodologies that could establish the etiology of respiratory disease–related mortality most efficiently in this setting.

### Hypothesis

Our primary hypothesis is that a high proportion of pediatric deaths are associated with viral etiology. The secondary hypothesis is that MITS can be successfully implemented in low-middle-resource countries to aid the cause-of-death diagnosis in hospital settings.

## Methods

### Study Setting and Overall Design

The Kenyatta National Hospital (KNH) is Kenya’s national referral and teaching hospital, located in Nairobi. The hospital has 4 general pediatric medical wards with 180-bed capacity, a 6-bed pediatric intensive care unit (ICU), a 20-bed general ICU, and a 20-bed high dependency unit that serve critically ill patients of all ages. The majority of its patients are referred from health facilities within Nairobi and the surrounding counties; however, tertiary referrals come from all over the country. In addition, the hospital has a 24-hour mortuary where bodies of deceased patients are refrigerated, as well as a facility for postmortem examination (autopsy).

The study enrollment is based on the prospective identification of children who were hospitalized for respiratory illness (based on case definition) and monitored throughout their hospital stay. Those who died were enrolled in the postmortem investigation portion of the study.

**Figure 1:**
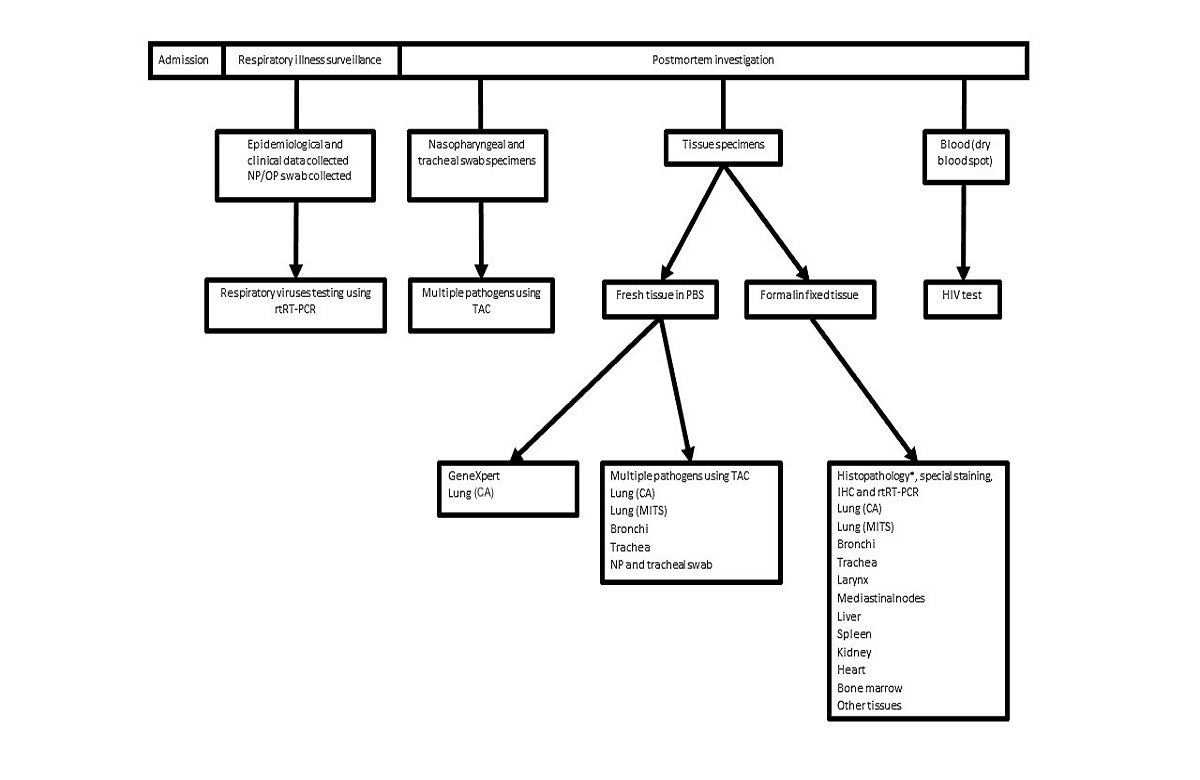
In-hospital surveillance and postmortem specimens and laboratory testing summary. CA: conventional autopsy; IHC: immunohistochemistry; MITS: minimally invasive tissue sampling; NP/OP: nasopharyngeal/oropharyngeal swabs; PBS: phosphate buffered saline; rtRT-PCR: real-time reverse transcription polymerase chain reaction; TAC: TaqMan array card. *The Kenyatta National Hospital/University of Nairobi did routine histopathology only. The Infectious Disease Pathology Branch laboratory did routine histopathology, special staining, IHC, and molecular tests.

A case of respiratory illness was defined as a hospitalized child aged 1-59 months, who presented with a cough or difficulty in breathing or if an attending physician diagnosed respiratory illness. At enrollment, we collected sociodemographic and clinical data and obtained nasopharyngeal and oropharyngeal swabs from consented patients. We then followed participants until discharge (or death) and abstracted additional data from the hospital files on the results of laboratory investigations, discharge diagnoses, and outcome of hospitalization. In addition, we included children who died of respiratory illness (per parental or guardian interview or physician’s assessment) at admission or soon after admission, regardless of enrollment while patients were still alive and collected as much data as available from hospital records. Trained grief counselors approached parents or guardians of deceased children and offered them counseling before seeking consent to perform postmortem examination. We performed MITS to obtain lung tissue specimens followed by the conventional autopsy to collect multiple tissue specimens. Tissues were examined through histopathology and tested using multiple laboratory techniques to identify etiologies potentially associated with respiratory disease–related death. Figure 1 summarizes these procedures and specimen collection and testing.

### Procedures

#### Premortem Enrollment of Hospitalized Respiratory Cases

We trained surveillance officers (clinical officers and nurses) on the study protocol and standard operating procedures (SOPs). We then assigned surveillance officers to each of the 4 pediatric wards of the KNH to provide 24/7 coverage for the identification of eligible children. Furthermore, surveillance officers visited the ICU or high dependency unit to enroll critically ill children who met the study case definition (described above) but who bypassed admission to the general ward.

For each eligible child, parents or guardians were approached to provide consent for their child’s enrollment in the study. Once consent was obtained, a structured enrollment questionnaire was administered to parents or guardians to collect demographics, duration of illness, care-seeking history for the current illness, symptoms and signs on admission, underlying chronic illnesses, indoor smoke exposure, and childhood vaccination status.

In addition, we abstracted from medical charts the recorded body temperature, oxygen saturation (assessed through pulse oximetry), anthropometric measurements on admission, and mother and child’s HIV status, and child’s antiretroviral therapy use, where available.

We followed enrolled children until discharge or death and collected additional data on the results of routine laboratory investigations conducted during hospitalization, radiological investigation reports, medications, and other treatment and discharge diagnoses (when available).

#### Premortem Nasopharyngeal and Oropharyngeal Specimen Collection

At enrollment, we collected nasopharyngeal or oropharyngeal swabs. We used a polyester-tipped, flexible, aluminum-shafted applicator (25-801D; Puritan) to collect nasopharyngeal specimens. The swab was inserted into one of the patient’s nostrils, gently pushed in parallel to the palate onto the nasopharyngeal wall from the edge of the nostril to the approximate level of the lower margin of the ear lobe. The swab was rotated 2-3 times and held in place for 3-5 seconds to absorb as much sample material as possible. The swab was removed and placed into a prelabeled cryovial containing 3 mL of viral transport media prepared at the Kenya Medical Research Institute (KEMRI) and the Centers for Disease Control and Prevention (CDC)-Kenya laboratory following the standard World Health Organization’s protocol [13]. The oropharyngeal swab was collected using a nylon-flocked, plastic-shafted applicator (Copan Diagnostics). Using a tongue depressor, the oropharyngeal swab was inserted to contact the posterior OP wall and rotated against the mucosal membrane for 3-5 seconds. The oropharyngeal swabs were then placed into the same prelabeled cryovial containing nasopharyngeal swab. The cryovial was stored and shipped to the KEMRI and CDC-Kenya laboratory in Nairobi at 2°C-8°C daily where it was stored at −80°C until testing.

#### Death, Body Handling and Preservation, and Consenting for Postmortem Examination

In the event of a death of a child with a respiratory illness, nurses wrapped the body in a clean sheet and labeled it. Technicians collected the body within 6 hours postmortem and transferred it to the mortuary where it was stored in a study-designated refrigerator at 4°C-8°C without embalming. A trained study counselor made a telephone call to the parents or guardians, scheduled an appointment for grief counseling, and explained the importance of conducting postmortem examination in establishing the actual cause and etiology of death. Written consent for a postmortem examination of the decedent’s body was obtained from parents or guardians following counseling.

For children who died before being enrolled into surveillance (eg, died at or soon after admission), we also contacted their parents or guardians and offered them grief counseling. We then interviewed them to determine if their child was eligible for enrollment. For parents or guardians whose children met our case definition, we asked for their consent for an interview about the child and to collect data on the child through medical chart abstraction and postmortem examination. If a parent or guardian declined to provide consent for postmortem examination, the body was removed from the designated refrigerator, embalmed, and stored in a nonstudy-designated refrigerator.

#### Postmortem Examination Data and Nasopharyngeal Specimen Collection

Study pathologists received a 1-week training on postmortem examination SOPs by 2 practicing autopsy pathologists with expertise in pediatric pathology from the University of Washington and Massachusetts General Hospital, and a lead pathologist from the Infectious Disease Pathology Branch (IDPB) of the CDC-Atlanta (Georgia, USA). The training, conducted at the KNH mortuary, ensured a consistent autopsy procedure and standardized specimen collection. Once consent for postmortem examination was obtained, the study pathologists were notified and arranged to perform the postmortem examination. The autopsies were performed as soon as possible but no more than 5 days after death.

We used standard data collection study tools to obtain data on the general examination findings, including the appearance of the body and anthropometric measurements. In addition, photographs of the face (for study identification purpose only) and of any abnormal physical features were taken. Furthermore, a nasopharyngeal swab specimen was obtained, as previously described, and placed it into a cryovial containing 3 mL of viral transport media.

#### Postmortem Collection of Specimen Using Minimally Invasive Tissue Sampling Techniques

**Figure 2:**
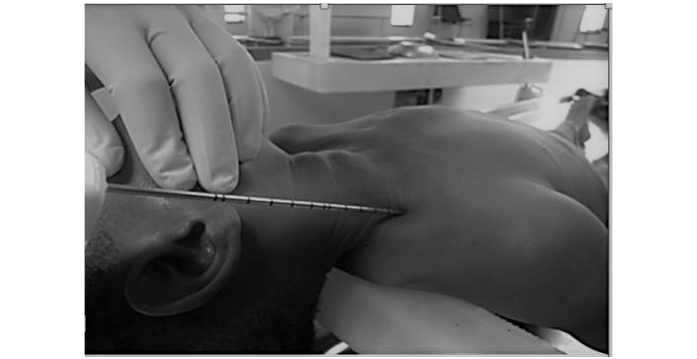
Minimally invasive tissue sampling of the lung at supraclavicular notch.

**Figure 3:**
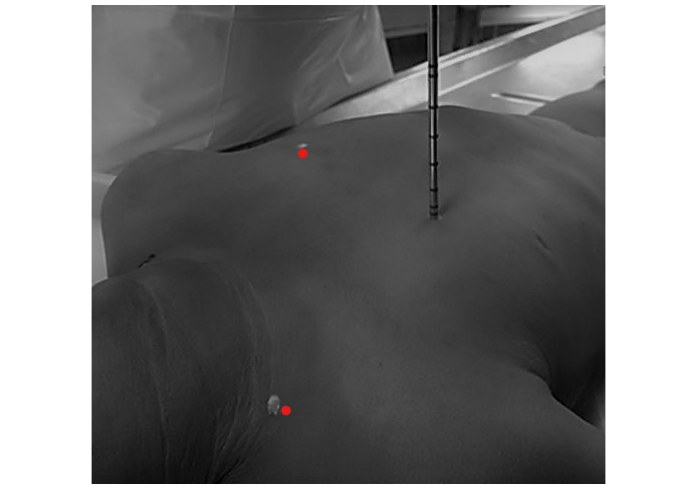
The fourth intercostal space midclavicular line, the red dot indicating the size and aspect of the biopsy needle point of entry.

The initial procedure was to sample the bilateral lungs through a needle biopsy. The skin was cleaned on the bilateral supraclavicular and anterior chest regions using alcohol swabs, until the swabs appeared clean, and the skin was allowed to air dry. Three sets of lung tissue specimens were separately collected by 11-gauge biopsy needle (ACECUT, Automatic Biopsy System, Ace-1152 the 11g 115mm 22mm, 2016 TSK Laboratory Europe B.V.). Each set of tissue contained specimens from 4 sites—bilateral 4^th^ intercostal spaces just lateral to the sternal border, and the supraclavicular region, in the midclavicular line, with the needle aimed inferiorly (Figures 2 and 3). The first set of lung tissue specimens collected was placed into a tissue jar containing phosphate buffered saline (PBS) and sent to the KEMRI and CDC laboratory in Nairobi for testing for multiple pathogens using TaqMan Array Cards (TAC), as described below. The second set of lung tissues was placed into labeled tissue cassettes, which were placed into a tissue jar containing formalin and sent to the University of Nairobi histopathology laboratory where 3 sets of tissue slides were prepared and later shipped to the 3 US-based pathologists for analysis. The third set was prepared similar to the second set of tissues and sent to the CDC IDPB laboratory in Atlanta for histology, special staining immunohistochemistry, and molecular tests. All laboratory tests performed are detailed below.

#### Postmortem Collection of Specimen Using Conventional Autopsy Methods

Using a sterile blade, a midline incision was made below the chin to the pubic symphysis. The skin was reflected to expose the internal organs. Using a sterile blade, a transverse incision was made in the anterior trachea below the thyroid process and a tracheal swab specimen collected from the internal trachea, using a Dacron-tipped swab, inserted, rotated, and held against the internal posterior tracheal wall collecting any secretions and placed into the same cryovial containing the previously collected nasopharyngeal swab. Using sterile instruments, the following tissue specimens were collected following standard procedures for autopsy: 4 pieces of trachea, 5 pieces of lung (one from each lobe of both lungs), 2 pieces of myocardium, 4 paired bronchial specimens (pair represents—one piece from each main bronchus), 4 mediastinal lymph node specimens, and 3 pieces from each of the liver, spleen, and bilateral kidneys; these tissues from each site were separately placed into a 10% formalin solution and fixed for 4-24 hours and, then, stored in 70% ethanol. Additional tissue specimens were collected from organs with visible or suspected pathology and placed separately into tissue jars containing formalin. Using aseptic techniques, blood was collected from the heart ventricles prior to dissection, and dried blood spots (DBS) were prepared by spotting whole blood on 5 circles of filter paper. The DBS cards were placed on a drying rack and left to dry overnight in a biosafety cabinet at room temperature (20°C). After drying, each card was placed in a glycine envelope. The glycine envelopes were subsequently packaged in a zip-lock plastic bag containing 4 desiccants and a humidity indicator card and stored at −20°C until testing. Any other fluids (pleural and pericardial space, abdominal cavity) and cerebrospinal fluid (CSF) were collected and placed into separate sterile specimen jars. The brain was examined using standard procedures. Microscopy of all organs and tissues was performed. At the end of the autopsy, a standard list of anatomic diagnoses based on the pathological findings was completed, as was a pathological cause of death based on the integration of the clinical and pathological findings.

### Laboratory Testing

#### Respiratory Virus Testing on Premortem Nasopharyngeal and Oropharyngeal Specimens

Combined nasopharyngeal or oropharyngeal specimens stored in –80°C freezers at the KEMRI and CDC laboratory in Nairobi were retrieved and nucleic acid purification performed for individual real-time reverse transcription polymerase chain reaction (RT-PCR) assays. Total nucleic acids were extracted from 100 µL aliquots of each sample using the MagNA Pure 96 DNA & Viral Nucleic Acid Kit in a MagNA Pure 96 instrument (Roche Inc), and the final material eluted in 100-μL buffer according to the manufacturer’s instructions.

#### Processing of Lung Tissue for TaqMan Array Cards Testing

Briefly, 30-mg frozen tissue was placed in a 2-mL microcentrifuge tube containing a 5-mm prechilled stainless steel bead (Sigma-Aldrich Chemie Gmbh, Munich, Germany) and incubated on dry ice for 15 minutes. The microcentrifuge tubes were placed in a TissueLyser LT Adapter (Qiagen Inc) and incubated at room temperature for 2 minutes, followed by the addition of 400 μL of MagNa Pure lysis buffer (Roche Diagnostics Corporation). The disruption and homogenization of the tissue were performed for 5 minutes at 50 Hz in TissueLyser LT (Qiagen Inc.). The subsequent total nucleic acid purification was performed using MagNA Pure 96 DNA & Viral Nucleic Acid Kit in a MagNA Pure 96 System (Roche Inc.) following the manufacturer’s instructions. Total nucleic acids were finally eluted in 100 µL and 46 µL tested in TAC as described below.

#### Multipathogen Testing Using TaqMan Array Cards

Total nucleic acids from nasopharyngeal or tracheal swabs, tracheal, bronchial, and lung specimens collected using MITS and conventional autopsy techniques were tested at the KEMRI/CDC laboratories in Nairobi using a TAC that tests for 21 pathogens as follows: 13 bacteria (*Bordetella pertussis, Chlamydia pneumoniae*, *Haemophilus influenzae all types, Haemophilus influenzae type B*, *Klebsiella pneumoniae*, *Legionella* spp, *Moraxella catarrhalis, Mycoplasma pneumoniae*, *Pseudomonas aeruginosa*, *Staphylococcus aureus*, *Streptococcus pneumoniae* and *Streptococcus pyogenes*, and *Mycobacterium tuberculosis)*; 1 parasite (*Pneumocystis jirovecii)*; and 15 viral pathogens (adenoviruses, coronaviruses 1,2,3,4, Enteroviruses, human metapneumovirus [hMPV], Influenza A, B viruses, parainfluenza 1,2,3,4, respiratory syncytial virus, and rhinoviruses). Assays were run on the ViiA-7 Real-Time PCR System with AgPaTH-ID One-Step Real-Time PCR Kits (Applied Biosystems). PCR Master Mix for each card included 1× RT-PCR buffer RT-PCR enzyme in the final volume of 100 µL reaction volume. We added 46 µL of nucleic acid extract to the master mix. Each run consisted of a negative control and a positive control for the first card of the day to be tested. A minimum of 3 cards were tested per day, with thermal cycling conditions as follows: 45°C for 10 minutes, 94°C for 10 minutes, and 45 cycles of 94°C for 30 seconds and 60°C for 1 minute. For each target, a TAC test result was considered positive if an exponential fluorescence curve was produced that crossed the assigned cycle threshold at ≤35.0. Previous studies have shown that specimens with TAC cycle threshold >35.0 may be less likely to be verified by sequencing of the amplicon [14,15].

#### Individual Real-Time Reverse Transcription Polymerase Chain Reaction

Individual real-time RT-PCR (IRTP) was carried out using AgPath-ID One-Step RT-PCR Reagents (Applied Biosystems). The TAC assays were compared with the cognate IRTP assays on 96-well plates under the same thermocycling conditions using the same PCR Master Mix and 5 µL of total nucleic acids as a template. Specimens were tested in duplicates for the presence of adenoviruses, hMPV, influenza A and B viruses, parainfluenza virus types 1-3, and respiratory syncytial virus. In addition, each clinical specimen was tested for the human ribonuclease protein gene to measure the nucleic acid integrity and confirm the sample adequacy. A qRT-PCR test result was considered positive if an exponential fluorescence curve was produced that crossed the assigned cycle threshold at <40.0.

#### Histopathology at Kenyatta National Hospital on Postmortem Specimens

Tissue specimens from the lungs, hilar lymph nodes, heart, kidneys, spleen, liver, brain, and other organs were fixed in a 10% formalin solution prepared in 0.9% buffered saline, for 24 hours prior to the slide preparation. Study-engaged pathologists reviewed the histology slides stained with hematoxylin and eosin (H&E) stain and documented observed pathological changes using structured data abstraction forms.

#### Tuberculosis Testing Using GenXpert

Lung tissues were sent to the KEMRI-CGHR TB laboratory for screening of *Mycobacterium tuberculosis* using GeneXpert (Figure 1); this is an off-label evaluation of tissue with the GeneXpert as the Food and Drug Administration Market Authorization only extends to testing of sputum in nonpediatric patients. Lung tissues were processed to remove all fats and 5 g of tissue diced into 0.5-cm pieces using a sterile scalpel blade. The diced tissues were placed in a homogenizing container and 10 mL sterile distilled water added. The specimen was homogenized for 3 minutes at 5000 rpm followed by decontamination using 10 mL of 0.5-M NaOH‐NALC. Thereafter, pellets were washed with 50 mL of 0.067-M PBS (pH 6.8) pelleted again and reconstituted in 2 mL PBS. Next, 1 mL of the suspended specimen was transferred to a 5-mL falcon tube and 3-mL Xpert MTB/RIF sample reagent added followed by vigorous shake 20 times. This was incubated at room temperature (24°C) for 15 minute with agitation every 5 minute. The liquefied sample was transferred to Xpert MTB/RIF cartridge (Cepheid) and tested following the manufacturer’s instructions.

#### HIV Testing

HIV antigen testing was done at the Kenya AIDS Vaccine Initiative laboratory on DBS collected during the autopsy. The Roche Amplicor HIV DNA PCR Kit was used for the PCR procedures, with some modifications. Briefly, a clean handheld punch (1/4 inch) was used to punch a disk (6 mm^2^) from the DBS into a 2 mL screw cap tube, and 1 mL of Roche Specimen Wash Buffer added. Then, DNA extraction, PCR amplification, and analysis by enzyme-linked immunosorbent assay were performed per the manufacturer’s recommendations [16]. Specimens were considered unequivocally positive if they had an optical density (OD) of 0.8 and negative if they had an OD <0.2 using an A450 filter. Specimens that had ODs >0.2 but <0.8 were considered indeterminate and retested.

#### Histopathology, Special Staining, Immunohistochemistry, and Molecular Tests

Minimally invasive lung autopsy tissues and full diagnostic pulmonary and extrapulmonary autopsy tissues fixed in a 10% buffered formalin solution were sent to the CDC IDPB where the tissues were paraffin-embedded, sectioned at 4 μm, and stained by routine H&E. For each case, the H&E from the minimally invasive lung autopsy tissues were evaluated and interpretations recorded prior to the pulmonary and extrapulmonary full diagnostic autopsy tissues. Based on the clinical information, TAC results and the evaluations from both sets of tissues histochemical stains, immunohistochemistry, and molecular testing were pursued, as summarized in Tables 1 and 2. Additional histochemical stains including Grocott’s methenamine silver (GMS), Lillie-Twort Gram (LT), Warthin–Starry silver (WS), Fontana-Masson, Prussian blue iron, and Ziehl–Neelsen acid-fast (ZN) were available for further testing as needed. Immunohistochemical assays were performed using a polymer-based colorimetric indirect immunoalkaline phosphatase detection system with colorimetric detection of antibody or polymer complex with Fast Red Chromogen (Thermo Fisher Scientific or Biocare Medical) with appropriate positive and negative control tissues tested in parallel for each assay. A multitude of antibodies targeting bacteria, fungi, and viruses were also utilized in some cases; these are summarized in Supplementary Tables 1 and 2. Molecular assays were performed in-house at IDPB or in conjunction with internal collaborators at the CDC. Optimized extraction protocols were used as previously published [17], and assay specifics are available upon request to IDPB.

**Table 1:** Outline of standard screening tests done at the Infectious Diseases Pathology Laboratory (IDPL), CDC-Atlanta.

Type of test	Screening tests^a^
Histological evaluation	Hematoxylin & Eosin
Histochemical stains	Lillie-Twort Gram
	Grocott's methenamine silver
Molecular viral panel	Influenza A and B viruses
	Parainfluenza viruses
	Respiratory syncytial virus

^a^Screening tests performed on the upper and lower airway tissue from complete diagnostic autopsy specimens and needle core tissues from minimally invasive tissue sampling.

**Table 2:** Outline of standard follow-up and confirmatory tests at the Infectious Diseases Pathology Laboratory (IDPL), CDC-Atlanta.

Category	Primary testing	Findings that trigger follow-up assay	Follow-up assay
Fungal	H&E^a^	Frothy intra-alveolar eosinophilic material	*Pneumocystis* spp IHC^b^
	GMS^c^	Crescentic fungal forms	*Pneumocystis* spp IHC
	H&E and H&E	Fungal hyphae and yeast	Fungal IHC assays^d^; Broad-range fungal PCR^e,f^
Viral	H&E and PCR	Phase I viral panel negative and interstitial pneumonitis	Additional PCR^g^; *Mycoplasma* spp; Rhinovirus; Human metapneumovirus
	GMS and IHC	Pneumocystis organisms	CMV^h^ IHC
	H&E	Compatible viral inclusions	CMV IHC
	H&E, GMS, IHC and clinical history	Concomitant diseases associated with immunosuppression	CMV IHC
Bacterial	H&E, LT^i^, and GMS	Gram-negative bacteria	*Klebsiella* /*Enterobacteriaceae* IHC^j^ (and confirmatory PCR)
	H&E and LT/GMS	Acute inflammation without bacteria on special stains	*Mycobacterium* spp IHC; *Streptococcus* spp IHC; *Staphylococcus* spp IHC; *Group B Streptococcus* spp IHC; *Group A Streptococcus* spp IHC; Warthin starry special stain
	H&E, LT/GMS, and IHC	Acute inflammation without bacteria on special stains or IHC	Pan eubacterial PCR
	LT	Gram-positive cocci	*Streptococcus* spp IHC; *Group A Streptococcus* spp IHC
	LT	Small Gram-negative coccobacilli	*Haemophilus influenzae* IHC

^a^H&E: Hematoxylin & Eosin.

^b^IHC: immunohistochemistry.

^c^GMS: Grocott's methenamine silver.

^d^Fungal IHC panel consists of one or more of the following based on H&E and GMS morphology and Gram stain characteristics when applicable: Polyfungal IHC, mucormycete fungus IHC, *Aspergillus* spp IHC, and *Candida* spp IHC.

^e^Broad-range fungal PCR performed by colleagues at the Mycotics Diseases Branch.

^f^PCR: polymerase chain reaction.

^g^Additional pneumonia-associated PCR assays based on H&E, clinical history, and TAC results.

^h^CMV: cytomegalovirus.

^i^LT: Lillie-Twort Gram.

^j^Polyclonal IHC known to cross-react with other *Enterobacteriaceae* bacteria.

### Data Review Process to Determine Cause of Death and Etiologic Determination

To determine the cause of death, clinical data, pathology findings (gross and histopathology reports), and results of laboratory investigations for each case were reviewed separately by a Kenya- and a US-based team of experts. The Kenya-based team (composed of 2 pediatricians, 3 pathologists and a medical epidemiologist) held monthly meetings to review data for each case. Based on the data reviewed, the team agreed on a preliminary cause of death. As local review of cases occurred close to real time, it did not include laboratory results from the other collaborating laboratories (in KEMRI-CDC and IDPB Atlanta) owing to delays in processing results.

A separate review was done by CDC IDPB, which reviewed histopathology findings and laboratory results, including special stains, immunohistochemistry, and other pathogen identification studies. Based on these data, they identified probable pathological (histopathological) diagnosis and suggested a pathogen etiology when possible.

**Figure 4:**
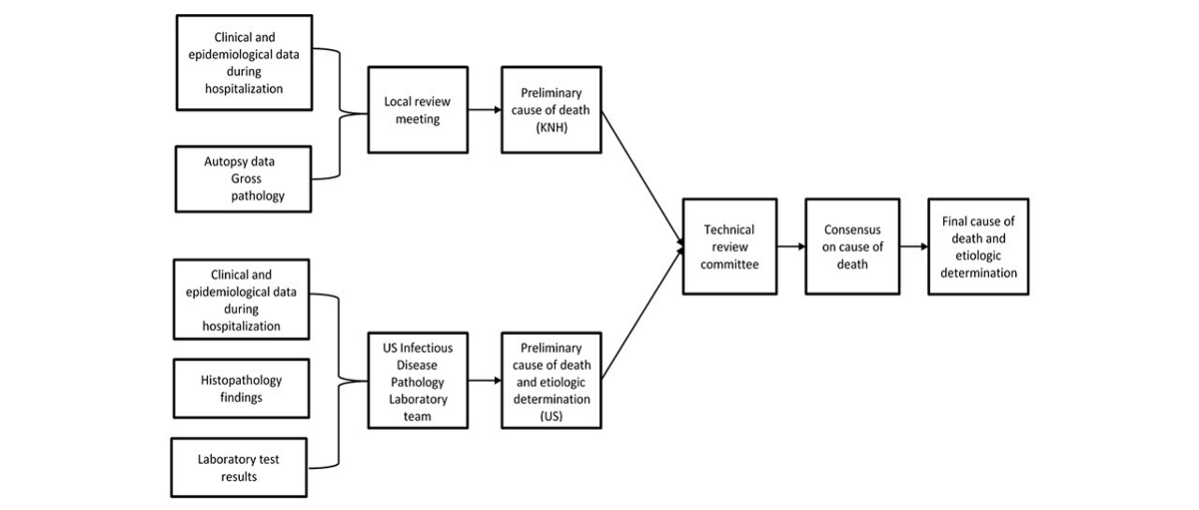
The algorithm to establish the final etiologic cause of death.

A final technical review committee was established comprising Kenya-based pediatricians and pathologists, laboratorians, epidemiologists, and US-based pathologists. This committee would review each case, assessing all clinical and postmortem findings, including laboratory investigation results, and agreed on a final cause of death and where applicable, identified etiologic pathogens associated with the cause of death (Figure 4). For cause of death determination, the consensus was sought, and the final classification followed the World Health Organization’s guidelines for medical certification of cause of death [18]. For etiology determination, the pathogen was considered on the basis of the presence in multiple tissues and from different tests (to exclude possible contamination or overgrowth), biological plausibility, and fit within the clinical presentation.

### Procedures for Comparing Findings From Minimally Invasive Tissue Sampling Techniques and Conventional Autopsy Techniques for Lung Specimens

Lung tissue specimens obtained through MITS and conventional autopsy techniques were sent decoded and unmatched to 2 US-based pathologists (from different institutions) for H&E diagnoses. The cases were reviewed separately, as slides were randomly labeled and not associated by case, independent of any clinical or pathological information. Then, the code was revealed, and specimens were matched and diagnoses compared between the Kenya-based team and the US-based team. Any discrepancy for each case and type of sample was resolved by consensus. In addition, for each case, we were able to compare pathogens detected by TAC in lung tissue collected using MITS to those collected using conventional autopsy.

### Dissemination of Study Findings

After each postmortem examination, Kenya-based pathologists provided feedback to parents or guardians on their gross examination findings. As histopathology and laboratory test results became available, parents or guardians were called and an appointment scheduled to relay this information. We participated in continuous medical education sessions targeting pathology and pediatric residents, their consultants, and pediatric ward nurses, where study cases were presented. Special emphasis was placed on children for whom the diagnosis was missed during the clinical evaluation and whose death could have been averted if diagnosis and right treatment was offered. Providing feedback to clinicians was an important strategy to engage hospital staff in our study, create awareness of clinical case definitions, and improve enrollment. In addition, results from our postmortem investigation were communicated back to parents and caregivers who accepted postmortem examination. All parents of children who died during the study period (whether they agreed to participate in the postmortem investigation or not) were later interviewed to assess whether they would have a change in perception toward the need for an autopsy and whether the use of only a MITS technique could have influenced their original reply to participation.

### Ethics Approval and Consent to Participate

The study protocol and procedures were reviewed and approved by the Institutional Review Board (IRB) at KEMRI (SSC no. 2692). We obtained ethical approval reliance from the IRB at CDC (6599) and KNH or University of Nairobi. The study was registered by the KNH’s Research & Programs Department (registration No. PEADS/0014/2014). Written informed consent was completed in either English or Swahili, depending on the parents or guardian’s language proficiency. Data collected from each case was identified using unique study identification numbers. We created a link log that was kept under lock and key and used for the sole purposes of linking patient data that were collected separately over time. No study participant was identified by name in any report or publication derived from information collected for the study. Furthermore, we paid for the cost of postmortem examination and associated histopathology and laboratory tests. We also paid for mortuary fees incurred for body storage at the mortuary for up to 10 days.

## Results

We have enrolled 945 pediatric cases of respiratory illness hospitalized in the KNH at the time of this submission; of those, 14.8% (140/945) died while in the hospital. We were able to successfully enroll 45.7% (64/140) deceased children for postmortem examination. The analysis and interpretation of laboratory results and writing of findings are expected in 2019-2020. We expect to have a manuscript on the comparison of MITS and conventional autopsy to detect pulmonary pathology among respiratory illness-related deaths; summary of the cause of deaths and associated etiologies among children who died from respiratory illness during hospitalization; factors influencing acceptance of postmortem examination of children; and will explore risk factors associated with in-hospital death in a resource-limited setting.

## Discussion

We describe a comprehensive approach to assess the causes of child respiratory mortality in a large urban Kenyan hospital. We brought together a diverse set of experts and applied a range of tests from basic histopathology to state-of-the-art immunohistochemistry and multipathogen molecular testing to detect a comprehensive range of pathogens in a wide range of tissues. We anticipate that study findings will add to the literature, as we will be able to compare the assessment of the cause of respiratory disease–related death based on the lung tissues collected using both MITS and conventional autopsy techniques. In addition, we will explore the use of multipathogen molecular-based diagnostic tools that can be used to support the cause of death determinations. Furthermore, we will be able to explore different pathogens simultaneously, including bacterial, fungi, mycobacterial, and viral agents and advise future studies in similar settings.

This study was not only designed to detect pathogens of clinical relevance in childhood respiratory disease–related deaths but also improve clinical care at the KNH and inform future studies of similar nature. Many studies have relied on the clinical assessment to determine the cause of death despite well-documented discrepancies between the clinical diagnosis and findings from autopsy [19-21]. Even with the improvement in diagnostic tools currently available to physicians in high-resource countries, an autopsy can aid in detecting up to 30% of causes of deaths missed during clinical evaluation [22,23]; this figure may be higher in resource-limited settings. However, autopsy rates throughout the world have declined [10]. The idea of using MITS to aid in postmortem investigations in remote areas where the capacity to perform a full autopsy is limited, and modern imaging techniques are not available, is not new [24]. MITS may improve acceptance rates among communities where autopsy procedures can be culturally less acceptable or feasible [25].

Investigation of postmortem-collected specimens may identify etiologic agents, which are not suspected antemortem, which is particularly relevant for respiratory illnesses, as many respiratory pathogens can cause similar syndromic presentations. For example, a retrospective study of adenoviruses in autopsies of pediatric patients who died of pneumonia in South China identified adenovirus by PCR in 9.1% (16/175) of lung tissues [26]. Another example is undiagnosed deaths due to influenza virus infection. This could be blamed on late care-seeking (where influenza viruses may no longer be detected in upper respiratory specimens) or lack of accurate diagnostic tests in the health care system. A study conducted among 1611 coronial autopsy cases in Western Australia found influenza in 8% of deceased children (age <10 years), none of whom were suspected of having influenza during clinical evaluation [27]. Moreover, it is difficult to ascertain the contribution of complications due to coinfections, such as secondary bacterial infection following influenza or other infections that could contribute to death, without the evaluation of lung or other tissues in the postmortem setting. Respiratory illness associated with viruses or fungi can be particularly difficult to detect as part of the routine clinical evaluation because of the limitations associated with antemortem sampling and the low sensitivity of available tests (eg, rapid antigen tests and blood culture) [28,29]. Another added benefit of postmortem investigation can be the possibility to assess a child’s neglect, abuse, and genetic conditions, which can go undetected until autopsy.

In this study, we enrolled patients to be followed from admission to final disposition (hospital discharge or death); this would allow us to integrate clinical history and management during hospitalization with the findings from postmortem investigation that can assist with defining the cause of death. Moreover, we will be able to compare pathogens detected during life with those detected postmortem to better understand the significance of findings when assessing causality.

As this study is hospital-based, our population may not be representative of deaths that occur in the community and may underestimate causes of death that rapidly progress in severity, leading to death prior to health care seeking. This is a limitation of a hospital-based study, especially in our case, where most patients are referred from other health care facilities. Additional problems could include contamination of postmortem tissue during collection (a special concern when using MITS techniques) [30], but the hospital setting may minimize this risk compared with studies conducted at the community. Moreover, percutaneous sampling methods could miss areas of pathology [31]. However, in this study, we will be able to link results from tissues collected using MITS with those collected during the full autopsy and assess MITS methods. Finally, postmortem investigative studies are associated with the immediate widespread cellular degradation after death facilitating bacterial translocation [32]. Bacteria, fungi, and endotoxins can cross the mucosal barrier of the gastrointestinal tract, and interpretation of multipathogen molecular diagnostic results can be complicated as these pathogens can multiply after death, masking the real etiologic agent. We hope to overcome the challenge of interpreting the relevance of pathogens detected using TAC testing by adding clinical history and information on the course of illness while a patient is alive and contrasting findings with that from different testing methodologies and tissue specimens to assist with the interpretation of etiology.

In conclusion, we describe important methodological procedures to assess the leading causes of pediatric respiratory disease–related deaths that can be adopted in similar hospital-based studies. In addition, this study would provide insights into the interpretation of results using multipathogen testing platforms and comparing different techniques (MITS and specimens through conventional autopsy) that can be used in studies or surveillance undertaken in other resource-limited settings.

## Abbreviations

CDC: Centers for Disease Control and Prevention
DBS: dried blood spots
H&E: hematoxylin and eosin
IDPB: Infectious Disease Pathology Branch
KEMRI: Kenya Medical Research Institute
KNH: Kenyatta National Hospital
LT: Lillie-Twort Gram
MITS: minimally invasive tissue sampling techniques
OD: optical density
PBS: phosphate buffered saline
PCR: polymerase chain reaction
PRESS: Pediatric Respiratory Etiology Surveillance Study
TAC: TaqMan Array Cards
